# Exploring Coagulase-Negative Staphylococci Diversity from Artisanal Llama Sausages: Assessment of Technological and Safety Traits

**DOI:** 10.3390/microorganisms8050629

**Published:** 2020-04-27

**Authors:** Annalisa Rebecchi, Francesco Miragoli, Constanza Lopez, Daniela Bassi, Cecilia Fontana

**Affiliations:** 1 DISTAS, Università Cattolica del Sacro Cuore, via Emilia Parmense 84, 29122 Piacenza, Italy; annalisa.rebecchi@unicatt.it (A.R.); daniela.bassi@unicatt.it (D.B.); 2 Biotechnology Research Centre (CRB), via Milano 24, 26100 Cremona, Italy; francesco.miragoli@unicatt.it (F.M.); constanzamaria.lopez@unicatt.it (C.L.); 3 Instituto Nacional de Tecnología Agropecuaria, Estación Experimental Famaillá, Famaillá 4172, Tucumán, Argentina

**Keywords:** llama meat, fermented sausages, coagulase-negative staphylococci, starter cultures, safety traits

## Abstract

Llama sausage is still produced following artisanal procedures, with the autochthonous microbiota being mainly responsible for the fermentation process. In this work, the taxonomical identification and technological-safety criteria of coagulase-negative staphylococci (CNS) isolated from two different productions of llama sausages (P: pilot and A: artisanal) were investigated. *Staphylococcus* (*S*) *equorum* and *S. saprophyticus* were the species most frequently found in P production, followed by *S. succinis* and *S. warneri;* a wider species variability was observed in A factory being *S. equorum, S. capitis, S. xylosus,* *S. pasteuri, S. epidermidis* and *S. saprophyticus* as the main identified species. The technological characterization of 28 CNS strains showed their ability to hydrolyze gelatin and tributyrin together with a relevant nitrate reductase activity. Phenotypic and genotypic approaches were conducted to investigate the main safety traits. Llama’s CNS strains exhibited weak decarboxylase and hemolytic activity and low biofilm production; additionally, no enterotoxin genes were detected. Correlation analysis between phenotypic and genotypic data showed low values for the biofilm parameters, while high correlation was observed for oxacillin, ampicillin, tetracycline and aminoglycosides resistance and their genetic determinants. Data obtained may contribute to broaden knowledge about the autochthonous strains of this poorly studied fermented product, thus helping to select an appropriate combination of potential starter cultures to improve llama sausage safety and quality.

## 1. Introduction

The Andean region in Northwestern Argentina exhibits singular environmental conditions that limit agricultural and breeding activities in several areas. Here, high altitudes allow the survival of native camelids of south America, of which guanaco (*Lama guanicoe*) and vicuna (*Vicugna vicugna*) are wild, while llama (*Lama glama*) and alpaca (*Lama pacos*) are domesticated [1]. These camelids are famous not only for the wool they produce, but also as a food source. Recently, the importance of llama meat has increased due to its high protein nutritional value and reduced fat and cholesterol contents [2]; therefore, the improving of the llama and alpaca breeding systems and meat production could represent a good opportunity for the Andean population [3]. In this regard, fermented sausages made with llama meat represent a product category strongly appreciated among the local and tourist consumers and their production can play an important role for the economy of these countries. Llama sausage is produced without the addition of starter cultures, following artisanal procedures with a non-standardized quality and safety of the final products. Llama sausage consists of a mix of llama meat, pork fat, sodium chloride, salt, sugarcane, spices and nitrite/nitrate, stuffed into a natural bovine casing and ripened for about 20–30 days. Several studies on meat fermentation demonstrated that Lactic Acid Bacteria (LAB) ensure the safety and stability of the foods by reducing the pH and producing bacteriocins, while coagulase-negative staphylococci (CNS), as the major group within the community of Gram-positive catalase-positive cocci (GCC+), are mainly responsible for the development of aroma as well as flavour by their proteolytic and lipolytic activity [4,5]. Furthermore, CNS are able to reduce nitrate in nitrite leading to the production of nitroso-myoglobin, which is important for the characteristic red color of these fermented products; additionally, superoxide dismutase and catalase activity of CNS are important to prevent lipid oxidation by the detoxification of superoxide radicals and the decomposition of hydrogen peroxide, respectively [6]. Recently, Fontana et al. [7] reported that in artisanal llama sausages, the fermentation process is conducted mainly by LAB species, particularly *Lactobacillus sakei*, constituting this native microbiota a source of biodiversity with potential technological and beneficial properties [7]. However, little information about the CNS group composition in fermented llama meat is available. In this context, to have a more detailed picture of this important microbial group, we performed a taxonomical identification followed by a technological characterization of CNS isolated from llama sausages. In addition, relevant safety criteria, such as antibiotic resistance, biogenic amine and biofilm formation, enterotoxins and hemolysin production were evaluated to better characterize and select potential autochthonous starter strains. Data obtained in this study could be important to improve the quality of preserving llama sausages, as well as gaining knowledge about the traditional traits of this niche product.

## 2. Materials and Methods

### 2.1. Sausage Production and Sampling

Gram-positive catalase-positive cocci (GCC+) were enumerated and isolated at different ripening times (0, 2, 4, 7, 14, 21, 28, 35 days) on Mannitol Salt Agar (MSA, Oxoid, Milan, Italy) during 48 h at 30 °C, from naturally fermented llama sausages produced in Northwest Argentina as reported by Fontana et al. [7]. Briefly, two batches of llama sausages were elaborated: one in a pilot plant (P) of the Meat Technology Center of Universidad Nacional de Jujuy, according to traditional techniques and the other in an artisanal small factory (A) located in San Pablo, Jujuy (Northwestern Argentina). Llama sausage formulation and physicochemical analysis was performed as described by Fontana et al. [7]. Presumptive GCC+ colonies were streaked on Baird-Parker agar (Oxoid, Basingstoke, UK) supplemented with egg yolk tellurite emulsion (Oxoid, Basingstoke, UK) (24 to 48 h at 37 °C) to select only the CNS [8]. After incubation, isolated colonies were used to test morphology, Gram and catalase reaction. A total of 120 colonies of presumptive CNS were grown overnight in Brain Heart Infusion (BHI, Oxoid, Milan, Italy) broth and stored at −80 °C after the addition of 20% of glycerol.

### 2.2. Molecular Identification and Typing

One-hundred and twenty colonies were screened by PCR (Polimerase Chain Reaction) using specific primers (Table S1) for conserved regions of the staphylococcal 16S rRNA gene [9]. DNA from colonies was obtained by a thermal protocol using a ready-to-use lysis solution (microLYSIS-PLUS^®^, Microzone Ltd., Haywards Heath, Sussex, UK), following the manufacturer’s instructions. To achieve the strain typing, the isolates were screened by Random Amplification of Polymorphic DNA (RAPD) according to Fontana et al. [10], using a set of primers described in Table S1. Amplification was performed in a GeneAmp PCR System 9600 thermocycler (Applied Biosystems). RAPD banding patterns were analyzed using Gel Compare software, Version 4.1. The Pearson correlation coefficient was used to calculate the similarity in the profile of bands and dendrograms were obtained by means of the unweighted pair group method using the arithmetic average (UPGMA) clustering algorithm. A coefficient of correlation of 95% was arbitrarily selected to distinguish the biotypes. One representative strain was analyzed by means of 16S rRNA gene sequence. Primers used for the PCR amplification and the reference for the reaction condition are described in Table S1. The PCR products were purified using the USB^®^ ExoSAP-IT^®^ PCR Cleanup (Affymetrix^®^, Santa Clara, CA, USA) following the manufacturer’s instruction and sequenced at the BMR Genomics (Padova, Italy). The taxonomical assessment was performed using BLAST (Basic Local Alignment Search Tool). All isolated strains were stored in Brain Hearth Infusion broth (BHI) (Oxoid, Milan, Italy) plus 20% (*v*/*v*) glycerol at –20 °C and deposited at Università Cattolica del Sacro Cuore collection with UC code ranged from UC7541 to UC7569.

### 2.3. Technological Characterization

#### 2.3.1. Proteolytic and Lipolytic Activity

The proteolytic activity on sarcoplasmic and myofibrillar pork proteins was assayed according to Mauriello et al. [11]. Proteolytic activity on BHI medium supplemented with 30 g/L of gelatin bacteriological (Merck, Darmstadt, Germany) was also investigated [12]. After 48 h of incubation at 37 °C, the presence of a clear zone surrounding the inoculated wells (measured in millimeters) indicated proteolytic activity.

Tributyrin agar (Oxoid, Milan, Italy), and BHI agar medium supplemented with 1% of Tween 20 were employed for the assessment of the lipolytic activities [12]. Plates were incubated at 37 °C for 3 days. The lipolytic activity was determined by the appearance of a clear halo surrounding the spots whose diameter was measured in millimeters.

#### 2.3.2. Nitrate Reductase Activity

Nitrate reductase activity was determined by the agar plate method on Yeast Tryptone Agar (YTA, Oxoid, Milan, Italy) supplemented with 1 g/l KNO_3_ as previously described [13]. The reduction of nitrate to nitrite is characterized by the presence of a red halo around the colony. The bacterial enzymatic activity was evaluated by the intensity of the color.

### 2.4. Phenotypic Traits and Safety Characterization

#### 2.4.1. Production of Biogenic Amines (BA)

The ability of the CNS strains to produce biogenic amines (histamine, cadaverine, tyramine and putrescine) was phenotypically evaluated following the protocol described by Bover-Cid and Holzapfel [14]. The strains were grown on a plate with Bover-Cid medium containing the corresponding precursor amino acid at 1% final concentration (L-histidine monohydrochloride, tyrosine disodium salt, L-ornithine monohydrochloride and lysine monohydrochloride), (Sigma-Aldrich, Steinheim, Germany). Strains were streaked in duplicate on the decarboxylase medium plates with and without amino acids (as control) and were incubated for 4 days at 37 °C, under aerobic conditions. The pH variation due to the BA production changes the medium color from yellow-green to light or dark purple.

#### 2.4.2. Hemolytic Activity

The α-β-and δ-hemolytic activity was determined following the protocol of Jeong et al. [15]. For the first activity, Tryptose Soya Agar (TSA, Oxoid, Milan, Italy) plates were added with 5% sterile horse blood (Oxoid, Milan, Italy), while for the β- and δ-hemolytic activity TSA plates were supplemented with 5% sterile sheep blood (Oxoid, Milan, Italy). Plates were aerobically incubated at 37 °C for 24 h and only for β hemolytic activity plates were subjected to cold shock at 4 °C for 12 h. Strains showing clear zone between 1 and 2 mm wide from the border of the colony were evaluated as weak activity, if the halo was less than 1 mm the hemolysis was considered as negative [16].

#### 2.4.3. Biofilm Production Capacity

Biofilm production was assessed in microtiter using the method reported by Kwasny et al. [17] and Asai et al. [18]. Briefly, the staphylococci strains were grown in Tryptone Soya Broth (TSB, Oxoid, Milan, Italy) with 3% NaCl and TSB with 1% of glucose and incubated at 37 °C, overnight. Appropriate dilutions of bacterial cultures were performed using the same fresh medium then added to a 96-well microtiter polystyrene plate and incubated at 37 °C under aerobic conditions. After 24 h, the adherent biofilm was washed twice with phosphate buffered saline (7 mM Na2HPO4, 3 mM NaH2PO4 and 130 mM NaCl at pH 7.4) to remove the non-adherent cells. Biofilm was dried at room temperature, fixed with ethanol (95%) and stained with crystal violet (1%) finally washed again three times with sterile water and air-dried. The biofilm growth was measured by microtiter plate reader (Multiskan FC, Thermo Scientific, Braunschweig, Germany) at 600 nm of absorbance. Biofilm formation was interpreted as negative if the optical density at 600 (OD_600_) was <0.1, moderate if OD_600_ was between >0.1– <1.0, and positive if OD_600_ was ≥ 1.0.

#### 2.4.4. Antibiotic Resistance (AR)

The phenotypic AR profile was investigated by the disc diffusion test [12], according to the guidelines of the National Reference Centre for Antimicrobial Susceptibility and internationally recognized standards of the Clinical and Laboratory Standards Institute (CLSI) [19]. Determinations were performed on Müeller–Hinton agar (Oxoid, Milan, Italy) using the antimicrobial susceptibility disks from Oxoid (Milan, Italy), as follows: cefoxitin (30 μg), gentamicin (10 μg), kanamycin (30 μg), erythromycin (15 μg), tetracycline (30 μg), ofloxacin (5 μg), clindamycin (2 μg), trimethoprim-sulfamethoxazole (1.25/23.75 μg), compound sulfonamides (300 μg), trimethoprim (5 μg) chloramphenicol (30 μg), rifampicin (5 μg), linezolid (30 μg), ampicillin (10 μg), cefotaxime (30 μg) and cephalexin (30 μg). Additionally, oxacillin was tested in broth by using microdilution methods as indicated by CLSI [19].

### 2.5. Molecular Characterization of Antibiotic Resistance and Virulence Determinants

PCR amplification of well-known structural genes associated with AR, staphylococcal enterotoxins and biofilm-related genes were performed using the primers and conditions reported in Table S1.

## 3. Results and Discussion

### 3.1. Typing and Identification of Staphylococci in Llama Sausages

In this study, 95 out of 120 colonies isolated from llama meat sausages (P and A productions) were taxonomically identified by molecular techniques as belonging to the *Staphylococcus* genus (44 and 51 from P and A plant, respectively). Plate count performed by Fontana et al. [7], on P and A Llama sausages production showed that the GCC+ group did not largely contribute to the total microbial population during llama sausages fermentation with final counts of 5.5 log CFU/g in P production and a decrease after 4 days to 4.0 log CFU/g in A production.

**Figure 1:**
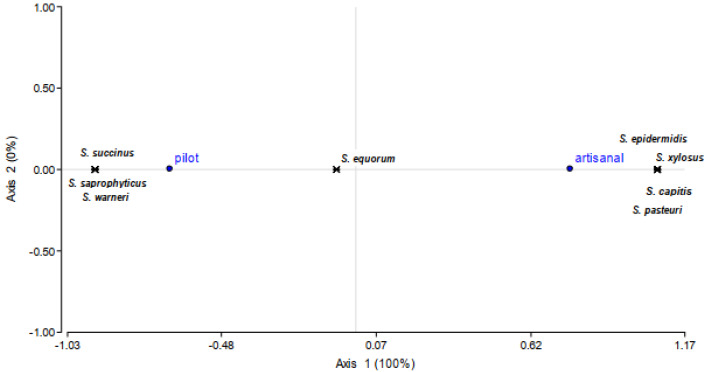
Correspondence analysis biplot of *Staphylococcus* (*S*) species and type of production (pilot or artisanal). Contribution to Chi-square is indicated in brackets.

The differentiation at the strain level was carried out by RAPD-PCR analyses using 3 different set of primers (Table S1), identifying 28 different biotypes (15 and 13 in P and A production, respectively). For each representative RAPD profile, one strain was selected and subject to 16S rRNA sequencing. *S. equorum* and *S. saprophyticus* were the species most frequently found in P production together with one strain belonging to *S. succinus* and *S. warneri* species. In contrast, a wider species variability was observed in the artisanal manufacturing where identified strains belonged to *S. equorum, S. capitis, S. xylosus, S. pasteuri, S. epidermidis* and *S. saprophyticus* species. A correspondence analysis showed the influence of the type of production on the CNS species development during the fermentation process (Figure 1).

**Figure 2:**
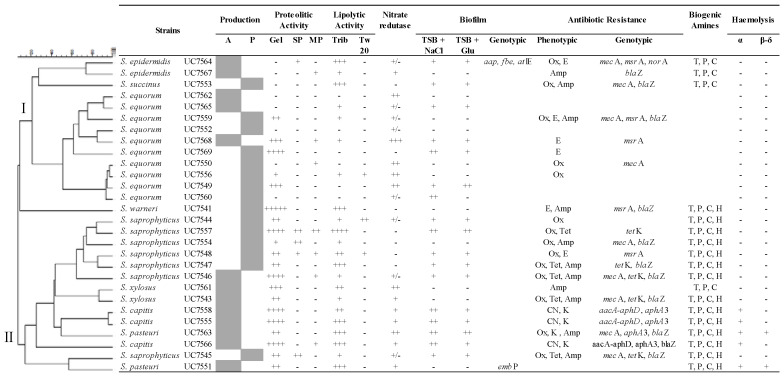
Cluster analysis of the profiles obtained by Random Amplification of Polymorphic DNA (RAPD) from the coagulase-negative staphylococci (CNS) strains isolated from the naturally fermented llama sausages from pilot plant (P) and artisanal (A) productions. Technological, functional and safety properties of isolates are reported in the table: Proteolytic activity (mm): Gelatin (Gel), Sarcoplasmic Proteins (SP); Myofibrillar Proteins (MP): no halo (-). 0.1–1 (+); >1–3 (++); >3–5 (+++); >5 (++++). Lipolytic activity (mm): Tributyrin agar (Trib); Tween 20 (Tw 20): (-).no halo (-). 0.1–1 (+); >1–3 (++); >3–5 (+++). Nitrate reductase negative (-); moderate red (±); evident red (+); deep red (++). Antibiotic Resistance: Ampicillin (Amp); Oxacillin (Ox); Tetracycline (Tet); Gentamicin (CN); Kanamycin (K); Erythromycin (E). Biogenic amines: Histamine (H); Tyramine (T); Putrescine (P); Cadaverine (C). Biofilm: Tryptone Soya Broth (TSB) with 3% NaCl and TSB with 1% Glucose (Glu): negative - (OD_600_ <0.1); moderate + (OD_600_ >0.1–<1.0) and positive ++ (OD_600_ ≥ 1.0). Hemolysis (mm): Tryptone Soy Agar added with horse blood (α); Tryptone Soya Agar added with sheep blood (β - δ); <1 negative (-); ≥1–<2 weak (+); >2 high (++).

To investigate their genetic relatedness, 28 biotypes were clustered by UPGMA; dendogram from Figure 2 shows the presence of two main clusters, one containing all *S. equorum* strains (10), *S. epidemidis* (2), *S succinis* (1) and the second cluster grouping all *S. saprophyticus* (7 strains), *S. capitis* (3), *S xylosus* (2), *S. pasteuri* (2). The solely *S. warneri* strain constitute a singleton. The predominance of *S. equorum* and *S. saprophyticus* species has also been described in other fermented dry sausage manufactured in Italy and in other Mediterranean regions [4,20]. The prevalence of *S. equorum* was observed in Spanish dry-cured salami [12,21,22] and in French dry sausage [23], whereas *S. saprophyticus* was the dominant species in artisanal Argentinean sausage [10].

### 3.2. Technological Properties

The technological properties were evaluated on the 28 staphylococci biotypes. Proteolytic and lipolytic are important activities for the sensory quality of fermented meat through release of various aromatic compounds and organic acids [20]. The proteolytic activity tested on three different protein sources was more evident on gelatin (71.4% of strains), especially by *S. capitis* strains. Only the 18% and 25% of strains showed weak proteolysis against sarcoplasmic or myofibrillar proteins, respectively. Contrary to our results, Cachaldora et al. [22] found higher proteolysis against myofibrillar and sarcoplasmic proteins and Landeta et al. [12] reported that only one CNS resulted positive to gelatinase activity assay. The lipolytic activity on tributyrin medium highlights a good activity (>1 mm of clear halo) in the 42.8% of the CNS strains analyzed, being *S. equorum* the less lipolytic strains (Figure 2). In contrast with our results, Jeong et al. [15] reported high tributyrin activity in *S. equorum* isolated from jeotgal, a Korean high-salt-fermented seafood, while Martin et al. [5] described *S. xylosus* and *S. epidermidis*, as the species showing high activity. Weak lipolytic activity on Tween 20, as lipid source, was observed only in two *S. saprophyticus* and one *S. equorum* strains (Figure 2). Similar results were found in a previous study [12] where none of the analyzed CNS strain were able to hydrolyze Tween 20.

The ability to reduce nitrate to nitrite contribute to the development of the sausage’s red color. In this study, all *S. capitis*, *S. pasteuri*, *S. xylosus* and most of the *S. equorum* strains were able to reduce nitrate to nitrite, whereas this activity was practically absent in *S. saprophyticus, S. succinis* and *S. warneri* (Figure 2). Similar results were reported by Sanchez Mainar et al. [24].

### 3.3. Safety Aspect

The capacity to produce biogenic amines is often present in CNS [6]. In this study, all CNS showed a positive decarboxylase activity (Figure 2) except for *S. equorum* strains. Our results are in accordance with Landeta et al. [12] that reported a low BA production in *S. equorum.* In addition, several authors observed a high decarboxylase activity in CNS from fermented meat products [22,25,26] with levels differing between species and strains [6,27].

Hemolytic activity is considered an undesirable property due to the ability to degrade erythrocytes. In this study, only *S. capitis* and S. *pasteuri* strains showed weak α-hemolytic activity; moreover, *S. pasteuri* performed β - and δ- activity too (Figure 2). Similarly, Zell et al. [16] and Jeong et al. [15] reported a moderate number of food associated CNS showing weakly haemolysis.

Bacterial biofilm constitutes a subject of considerable interest in food hygiene, particularly, in food processing environments [28]. For this reason, it is important to test this property in potential starter culture strains. Although, the formation of the biofilm is not a characteristic directly connected to virulence [29], this ability could confer more cell resistance to environmental stress than the planktonic cells [15]. To evaluate the ability of the strains to form biofilm is important to perform firstly the optimization of the media conditions [17,30]. Our results showed that the number of CNS strains able to produce biofilm was similar when 3% NaCl or 1% glucose was added to TSB medium (60.7% and 57.1%, respectively), while the biofilm amount produced was increased by the presence of NaCl in the media, with minor exceptions (Figure 2). Potter et al. [30] observed that the glucose addition to the culture medium could inhibit or have no effect on biofilm production by CNS strains, while the NaCl addition induced the biofilm production in some strains. In accordance with our results, Khusro et al. [29] and Landeta et al. [12] observed that the biofilm formation of CNS is a strain-specific characteristic, regardless of species. Analyzing the biofilm genetic profile, none of the CNS strains tested in this study was positive for the *ica*A and *bap*2 genes (Figure 2). *S. epidermidis* UC7564, a positive biofilm producer, harbored three out of six determinants tested (*fbe*, *atl*E and *aap*), while the *emb*P gene was only found in the negative biofilm producer *S. pasteuri* UC7551 (Figure 2).

The enterotoxigenic potential of CNS has always been a subject of controversy [6,31]. The CNS species have generally been regarded as non-pathogens, but in the recent years they have been involved in some case of food poisoning or human disease due to enterotoxins production [15]. In our study, no enterotoxin genes were detected in tested CNS strains. In accordance with our results, low frequency in the occurrence of the enterotoxin genes among CNS from food was also reported by several authors [13,31,32,33]. On the contrary, Nunes et al. [33] observed a high incidence of *seb, sec* (57%) and *sea* (50%) determinants among CNS strains isolated from commercial and artisanal salami.

The phenotypic and genotypic AR profiles are summarized in Figure 2. Most of the CNS strains (78%) showed at least one antibiotic resistance: oxacillin resistance was observed in 50% of biotypes following by ampicillin (39.3%), erythromycin (21.4%), tetracycline and kanamycin (both 14.3%) and gentamicin (10.7%). Five out of ten *S. equorum* strains and *S. pasteuri* UC7551 were sensitive to all antibiotics considered in this study. Moreover, multidrug resistance (MDR) was observed in more than half (53.6%) of CNS analyzed, with at least one strain of each species showing resistance to two or three antibiotics (Figure 2), especially in *S. saprophyticus* strains. Nevertheless, *S. equorum* was the species more sensitive to the tested antibiotics, *S. equorum* UC7559 showed to be resistant to three antibiotics. Resistances to penicillin, erythromycin and tetracycline are commonly found in food-associated CNS [6], varying in incidence, with some strains showing a complete lack of resistance, whereas others are resistant to several antibiotics [16,32,33,34]. In general, *S. saprophyticus* turned out to be the species with the highest percentage of antibiotic-resistant phenotypes among CNS isolated from meat products [32,35]. In accordance with our results, the low AR prevalence in *S. equorum* was reported by Landeta et al. [12].

The genotypic AR profile, summarized in Figure 2, shows that all the ampicillin-resistant strains harbored the *bla*Z gene, being the most common determinant detected among CNS isolated from llama sausage. Only 9 out of 14 methicillin/oxacillin resistance strains presented *mec*A gene. Five out of six erythromycin resistant CNS harbored *msr*A gene, known to code for macrolide efflux, while none of them carried *erm*A, *erm*B and *erm*C determinants. Regarding to tetracycline resistance, all four strains owned *tet*K gene, but none of the isolates carried *tet*M and *tet*L genes. Finally, concerning to the aminoglycosides resistance, *S. capitis* strains harbored *aacA-aphD* and *aph*A3 determinants, while *S. pasteuri* UC7563 only *aac*A-*aph*D gene.

**Figure 3:**
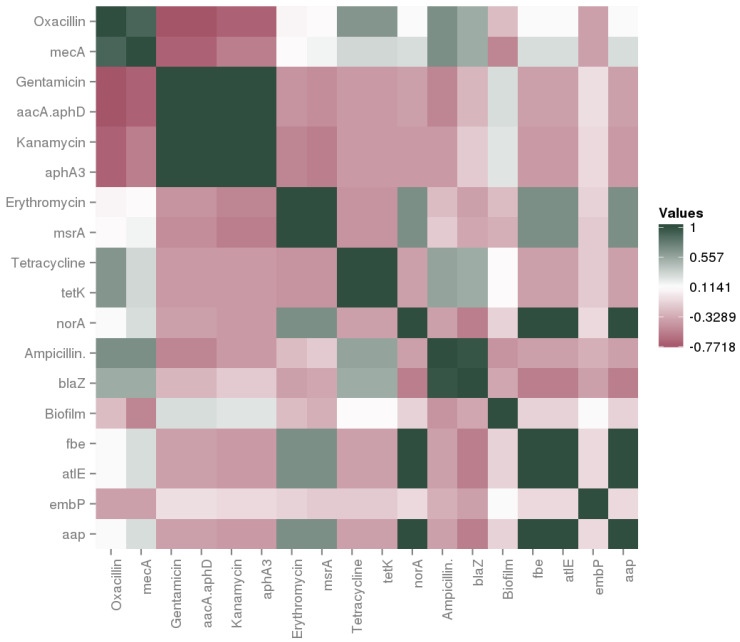
Correlation matrix of phenotypic and genotypic features regarding antibiotic resistance and bioflm formation ability.

Figure 3 shows a correlation analysis based on the phenotypic and genotypic results for AR and biofilm production.

High correlation (> 0.5) was found between phenotypic AR for oxacillin, ampicillin, tetracycline and aminoglycosides with the presence of their respective genetic determinant *mec*A, *bla*Z, *tet*K and *aacA-aphD* and *aph*A3. Our results are in contrast to those published by Resch et al. [16] and Fijałkowski et al. [36], who did not detect the *mec*A gene in any of the phenotypically resistant CNS, which may be due to an intrinsic resistance or to the presence of unknown resistance genes. In accordance with our results, Marty et al. [37], Landeta et al. [12], and more recently, Pyzik et al. [38], reported the wide spread of the *bla*Z gene among CNS due to the improper use of β-lactams in animal food. Regarding the resistance to tetracycline, several authors reported that *tet*K was the most prevalent gene among tetracycline CNS resistant from fermented meat, even if *tet*L and *tet*M determinants were also detected [37,38]. On the other hand, high correlation among erythromycin resistant strains with *msr*A gene was found (Figure 3). Although, high incidence of *msr*A gene has also been described in CNS isolated from ready-to-eat food of animal origin [39], Rebecchi et al. [35] reported that *erm*C gene was the most frequently present among CNS erythromycin resistant.

In this study, no correlation between phenotypic ofloxacin resistance and the presence of *nor*A gene was observed; in fact, this genetic determinant was detected only in *S. epidermidis* UC7564. Chajecka-Wierzchowska et al. [39] observed similar results for cefoxitin, reporting that this difference may be due to the presence of a “silent gene”. The *nor*A gene in *S. aureus* codifies for the efflux pumps, providing resistance to a variety of fluoroquinolones [40]. Methicillin resistance is often accompanied by resistance to other antimicrobial agents such as fluoroquinolones [41]. However, in this study, low correlation between *mec*A and *nor*A genes was found (Figure 3). Artini et al. [42] reported that all genetic *nor*A-positive CNS strain from cheese were also phenotypic ofloxacin resistant and no strain carrying both *mec*A and *nor*A genes was observed.

Concerning biofilm capacity, phenotypic and genotypic results led us to conclude that there was no relationship between the presence of the tested genes and the phenotypic profile in the analyzed strains. Similar results were obtained by Landeta et al. [10] and Potter et al. [29], so that we can suppose that the genes tested in this study are not essential for biofilm formation and thus the existence of other genes involved in biofilm production could be possible.

## 4. Conclusions

To our knowledge, this is the first report on the technological and safety traits of CNS involved in the manufacturing process of llama fermented sausages. In this study, CNS species development during the fermentation process was mainly influenced by the production technology applied (artisanal or pilot plant). Based on the technological and safety profile obtained from the autochthonous CNS, the most relevant technological performance was observed in *S. equorum* UC7562 and UC7549 strains. These microorganisms, in combination with the autochthonous LAB strains, could be considered as potential candidates for the starter formulation to be used by the local meat industry, thus preserving the unique features of llama fermented sausages and building confidence towards their safety and quality.

## Supplementary Materials

The following are available online at https://www.mdpi.com/2076-2607/8/5/629/s1, Table S1. Primer used in this study.
