# Supplementary material for: Exploring Coagulase-Negative Staphylococci Diversity from Artisanal Llama Sausages: Assessment of Technological and Safety Traits
Source: Microorganisms. 2020 Apr 27;8(5):629. doi: 10.3390/microorganisms8050629 (PMC7284484; doi:10.3390/microorganisms8050629)
Supplement: Supplementary file 1 [file microorganisms-08-00629-s001.zip › Table S1 Supplementary material.docx]

**Table 1.** Primer used in this study.

| Target gene | Forward primer (5’- 3’) | Reverse primer (5’- 3’) | Product (bp) | References |
| --- | --- | --- | --- | --- |
| 16S rRNA  *Tstag765/TstaG422* | GGCCGTGTTGAACGTGGTCAAATCA | TIACCATTTCAGTACCTTCTGGTAA | 370 | Morot-Bizot et al., 2004 |
| Universal primers | GAGAGTTTGATCCTGGCTCAG | CTACGGCAACCTTGTTACGA | 1560 | Di Cello and Fani, 1996 |
| RAPD |  |  |  |  |
| RAPD5 | AACGCGCAAC |  |  | Johansson et al., 1995 |
| XD9 | GAAGTCGTCC |  |  | Fontana et al., 2005 |
| RAPD2 | AGCAGCGTGG |  |  |  |
| Antibiotic Resistance |  |  |  |  |
| *blaZ* | ACTTCAACACCTGCTGCTTTC | TGACCACTTTTATCAGCAACC | 173 | Martineau et al., 2000 |
| *mecA* | AACAGGTGAATTATTAGCACTTGTAAG | ATTGCTGTTAATATTTTTTGAGTTGAA | 174 |  |
| *tetK* | TATTTTGGCTTTGTATTCTTTCAT | GCTATACCTGTTCCCTCTGATAA | 1159 | Trzcinski et al., 2000 |
| *tetL* | ATAAATTGTTTCGGGTCGGTAAT | AACCAGCCAACTAATGACAATGAT | 1077 |  |
| *tetM* | GAACTCGAACAAGAGGAAAGC | ATGGAAGCCCAGAAAGGAT | 740 | Olsvik et al., 1995 |
| *aac(6’)-Ie+aph(2’)* | CCAAGAGCAATAAGGGCATACC | CACACTATCATAACCATCACCG | 347 | Schmitz et al., 1999 |
| *ant(4 )-Ia* | CTGCTAAATCGGTAGAAGC | CAGACCAATCAACATGGCACC | 172 |  |
| *aph(3’)-IIIa* | CCGCTGCGTAAAAGATAC | GTCATACCACTTGTCCGC | 600 | Perreten et al., 2005 |
| *ermA* | TCTAAAAAGCATGTAAAAGAA | CTTCGATAGTTTATTAATAATAGT | 645 | Sutcliffe et al., 1996 |
| *ermB* | GAAAAGGTACTCAACCAAATA | AGTAACGGTACTTAAATTGTTTAC | 639 |  |
| *ermC* | TCAAAACATAATATAGATAAA | GCTAATATTGTTTAAATCGTCAAT | 642 |  |
| *msrA* | GGCACAATAAGAGTGTTTAAAGG | AAGTTATATCATGAATAGAT TGTCCTGTT | 940 | Ross et al., 1990 |
| *norA* | GCTATTATCGGTGGAGGCGTG | TTTGCTTCTTTACGGCGTGAC | 435 | Juárez-Verdayes et al., 2012 |
| Enterotoxin |  |  |  |  |
| *seA* | ACGATCAATTTTTACAGC | TGCATGTTTTCAGAGTTAATC | 544 | Rosec and Gigaud, 2002 |
| *seB* | GAATGATATTAATTCGCATC | TCTTTGTCGTAAGATAAACTTC | 416 |  |
| *seC* | GACATAAAAGCTAGGAATTT | AAATCGGATTAACATTATCCA | 257 |  |
| *seD* | TTACTAGTTTGGTAATATCTCCTT | CCACCATAACAATTAATGC | 334 |  |
| *seE* | ATAGATAAAGTTAAAACAAGCAA | TAACTTACCGTGGACCC | 170 |  |
| *seG* | GTTAGAGGAGGTTTTATG | TTCCTTCAACAGGTGGAGA | 198 | Bania et al., 1996 |
| *seH* | CAACTGCTGATTTAGCTCAG | CCCAAACATTAGCACCA | 173 |  |
| *seI* | GGCCACTTTATCAGGACA | AACTTACAGGCAGTCCA | 328 |  |
| *seJ* | GTTCTGGTGGTAAACCA | GCGGAACAACAGTTCTGA | 131 |  |
| *seK* | GGAGAAAAGGCAATGAA | TAGTGCCGTTATGTCCA | 516 |  |
| *seL* | CGATGTAGGTCCAGGA | TTCTTGTGCGGTAACCA | 369 |  |
| *seM* | CATATCGCAACCGCTGA | TCAGCTGTTACTGTCGA | 148 |  |
| *seN* | GGCAATTAGACGAGTCA | ATCGTAACTCCTCCGTA | 237 |  |
| *seO* | GTCAAGTGTAGACCCTA | TGTACAGGCAGTATCCA | 288 |  |
| *seP* | TCAAAAGACACCGCCAA | ATTGTCCTTGAGCACCA | 396 |  |
| *seQ* | GGAATTACGTTGGCGAA | AACTCTCTGCTTGACCA | 330 |  |
| Biofilm |  |  |  |  |
| *bap2* | GAGCCAGATAAACAACAAGAAG | CATGCTCAGCAATAATTGGATC | 598 | Tremblay et al., 2013 |
| *aap* | GAAGCACCGAATGTTCCAACTATC | AGTTGGCGGTATATCTATTGTA | 289 |  |
| *icaA* | CTGTTTCATGGAAACTCC | TCGATGCGATTTGTTCAAACAT | 200 |  |
| *fbe* | CTACAAGTTCAGGTCAAGGACAAGG | GCGTCGGCGTATATCCTTCAG | 273 |  |
| *atlE* | CAACTGCTCAACCGAGAACA | TTTGTAGATGTTGTGCCCCA | 682 |  |
| *embP* | AGCGGTACAAATGTCAAT | AGAAGTGCTCTAGCATCATCC | 455 |  |
